# Cohort Profile: The Ecuadorian National Birth and Infant Nutrition cohort

**DOI:** 10.1093/ije/dyag221

**Published:** 2026-10-04

**Authors:** Juan Carlos Palacios Mora, Monsermín Gualán, Irina Chis Ster, Natalia Romero-Sandoval, Philip J Cooper

**Affiliations:** School of Medicine, Universidad Internacional del Ecuador UIDE, Quito, Ecuador; School of Medicine, Universidad Internacional del Ecuador UIDE, Quito, Ecuador; School of Health and Medical Sciences, City St. George’s University of London, London, United Kingdom; School of Medicine, Universidad Internacional del Ecuador UIDE, Quito, Ecuador; School of Medicine, Universidad Internacional del Ecuador UIDE, Quito, Ecuador; School of Health and Medical Sciences, City St. George’s University of London, London, United Kingdom

**Keywords:** birth cohort, administrative data linkage, child growth, health inequalities, Ecuador

Key Features

- **The Ecuadorian National Birth and Infant Nutrition (ENBIN) cohort** is a national birth cohort including >1 million children born in Ecuador (2020–2023), with longitudinal follow-up to age 2 years using linked administrative data. It enables analysis of the social and biological determinants of early-life nutrition and health inequalities at national scale.
- **Participants** include all children born in Ecuador between 2020 and 2023 who are registered in the National Civil Registry or attend public health services. Birth registration coverage exceeds 95% (i.e. over 1 million children); anthropometric follow-up is available for approximately 76% of children.
- **Follow-up** is conducted continuously from birth to 2 years of age using individual-level administrative records from health, civil registration, and social protection systems, allowing the study of growth trajectories and time-varying exposures.
- **Integrated data** on demographics, birth conditions, anthropometry, socioeconomic status, maternal characteristics, health service utilization, and exposure to social protection programmes.
- **Data access** is managed by the Social and Environmental Determinants of Health Inequalities Unit (SEDHI) housed at Universidad Internacional del Ecuador (UIDE), and data are available for collaborative research subject to data access agreements.

## Why was the cohort set up?

Child survival and development are strongly shaped by social conditions including socioeconomic disadvantage, rural residence, maternal education, and ethnic marginalization [1–3]. Childhood undernutrition remains a major contributor to mortality and morbidity, while stunting during the first 1000 days has long-term consequences for cognitive development, educational attainment, productivity, and intergenerational poverty [4–7].

Ecuador is an ethnically diverse Andean country with substantial income inequality and deprivation. Despite sustained reductions in under-5 mortality and recent improvements in child growth, stunting remains high for an upper middle-income country (17.5% among under-5s in 2024) and is markedly higher among Indigenous children (34.5%) [8–10]. Ecuador also faces a double burden of malnutrition, with childhood overweight/obesity coexisting with chronic undernutrition [11–13].

Until recently, national monitoring of child nutrition in Ecuador relied primarily on periodic cross-sectional surveys, including the National Survey on Child Malnutrition (Encuesta Nacional sobre Desnutrición Infantil—ENDI) [8]. While these surveys provide nationally representative prevalence estimates, they do not permit longitudinal analyses. Previous data systems did not support the integration of birth characteristics, early growth trajectories, socioeconomic conditions, and exposure to social protection programmes within a unified longitudinal framework; at present, no metadata infrastructure exists to facilitate this integration. This limited the ability to examine how biological and social determinants jointly shape early-life nutrition, and how shifts in service coverage and inequality translate into changes in growth outcomes.

In 2021, Ecuador launched the National Strategy ‘Ecuador Grows Without Child Malnutrition’ (ENECSDI) [Estrategia Nacional Ecuador Crece sin Desnutrición Infantil] to strengthen intersectoral coordination in tackling undernutrition and improving monitoring through the integration of activities across the National Civil Registry of Ecuador (NCRE), the health sector, social protection programmes, and the National Poverty Registry. The national administrative tracking system for pregnant women and children younger than 2 years of age implemented under ENECSDI is the ‘Sistema Unificado y Universal de Seguimiento Nominal’ (SUUSEN: Unified and Universal Nominal Tracking System), an individual-level administrative data platform that anonymizes and links demographic, social, and clinical data. The establishment of SUUSEN created a unique opportunity to construct a population-scale longitudinal birth cohort based on linked administrative data.

The Ecuadorian National Birth and Infant Nutrition (ENBIN) birth cohort was established to harness this opportunity. ENBIN is designed to enable analysis of early-life social and biological determinants of child nutrition at national scale, to examine inequalities in birth and growth outcomes, and to provide a platform for future life-course linkage with mortality, educational attainment, hospital admissions, disease registries, social security records, and other administrative databases. Discussions are ongoing regarding the feasibility of linking ENBIN to Ministry of Health and social insurance datasets to extend follow-up beyond early childhood. ENBIN complements nationally representative survey data by providing a dataset built with longitudinal administrative records for understanding the determinants of child health and nutrition in Ecuador.

## Who is in the cohort?

The ENBIN birth cohort is population-based and uses anonymized administrative data derived from the SUUSEN tracking system. ENBIN includes children born between 1 January 2020 and 31 December 2023. ENBIN therefore comprises four closed annual birth cohorts (2020–2023), with longitudinal follow-up from birth to 24 months conditional on availability of administrative records.

The dataset comprises linked records from five national administrative data sources: birth registration (NCRE), digital birth notifications (REVIT), prenatal and well-child consultations (Ministry of Public Health records), cash transfers, childcare, home-visiting (Ministry of Human Development programme registries), and the poverty score derived from a proxy means test (Social Registry [SR]). Records are linked within SUUSEN using encrypted personal identification numbers assigned at birth registration. For research purposes, identifiers were irreversibly anonymized prior to data transfer. Deterministic linkage based on unique personal identifiers is near-complete for registered births, achieving a match rate exceeding 99%; linkage to follow-up data depends on subsequent contact with public health services.

Initial inclusion into the birth cohort is based on two primary data sources: (i) birth registration in the NCRE, which covers more than 95% of all children younger than 2 years of age nationally [8]; and (ii) postnatal anthropometric data recorded during ‘well-child’ consultations at the Ministry of Public Health primary care centres, estimated to serve approximately 76% of the population [8]. Within the NCRE, digital birth notifications (REVIT) are available for 88.3% of registered births. NCRE registry provides basic demographic information, while REVIT provides detailed information on birth conditions.

Although birth registration coverage is high, anthropometric follow-up depends on contact with public health services and declines with age. Differential service engagement, migration, private-sector care, and administrative recording practices may therefore affect representativeness, particularly at older ages.

Comparison with the nationally representative ENDI survey indicates that children living in urban coastal areas and those self-identifying as Indigenous, Afro-Ecuadorian, or Montubio may be under-represented within ENBIN, whereas children living in rural areas are relatively over-represented. These differences may reflect differential engagement with public-sector services, internal migration, use of private healthcare, and differences in the measurement and administrative classification of ethnicity and residence. Because both administrative capture and subsequent follow-up may be socially patterned, analyses of inequalities should evaluate potential selection and consider appropriate weighting and sensitivity analyses.

A total of 1 019 789 births registered between 1 January 2020 and 31 December 2023 are included in the cohort. Of these, 899 981 (88.3%) children had a digital birth transcript. Figure 1 shows the flow of children in each birth year contributing follow-up data across 6-month age intervals (0–5, 6–11, 12–17, and 18–23 months, inclusive). Complete 2-year follow-up is available for children born in 2020 and 2021; follow-up is progressively incomplete for those born in 2022 and 2023 because data were extracted in March 2024.

**Figure 1:**
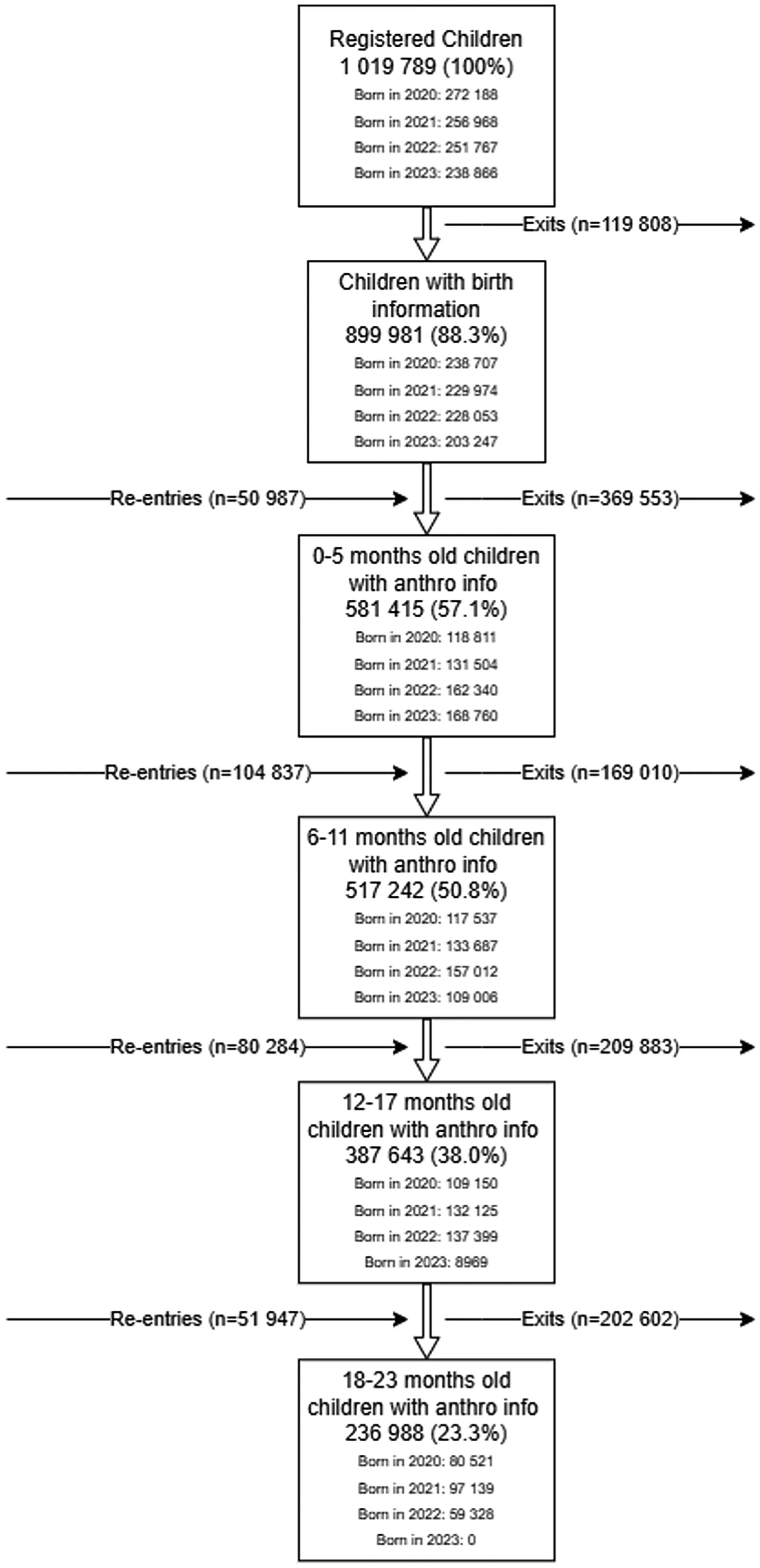
Participant flow diagram for ENBIN cohort. Due to the March 2024 data cut-off, follow-up data at older ages for children born more recently is missing—for children born in 2022, follow-up is available only for those born in the first months of the year at 18–23-months, while for children born in 2023, only those born in the first 3 months have follow-up data for the 12–17-month period. Age groups are inclusive.

For comparison, characteristics of children under 2 years from the nationally representative National Survey on Child Malnutrition (ENDI) 2022–2023 survey [8] are shown alongside ENBIN data in Table 2. A comprehensive analysis of malnutrition using the ENDI survey has been published recently [13]. Differences between ENBIN and ENDI estimates reflect contrasts between administrative and household-based data collection systems and highlight potential under- or over-representation of specific geographic and ethnic subgroups within routine administrative records. These differences may be partly addressed using external population benchmarks, including the 2022 Census, to construct post-stratification weights for population-level analyses [14].

**Table 2:** Comparison of characteristics of ENBIN cohort children by birth year with those of children under 2 years from a representative national survey (ENDI, 2022–2023).^a^

Characteristic	Born in 2020 (*n* = 272 188)	Born in 2021 (*n* = 256 968)	Born in 2022 (*n* = 251 767)	Born in 2023 (*n* = 238 866)	ENDI^b^ (*n* = 8264)
**Sex**					
Male	51.2%	51%	50.9%	51%	51.2%
Female	48.8%	49%	49.1%	49%	48.8%
**Ethnicity**					
Indigenous	6.5%	6.7%	7%	6.7%	8.8%
Black	1.5%	1.5%	1.5%	1.6%	5%
Montubio	1.8%	2.1%	1.9%	2%	5.5%
Mestizo	89.4%	89%	89%	89.2%	78.6%
Other	0.8%	0.7%	0.6%	0.5%	2.1%
**Poverty status (SR score)** ^c^					
Not poor	18.2%	14.9%	15.6%	15.6%	NA
Poor	27.7%	29%	29.2%	28.9%	NA
Extremely poor	12.3%	18.7%	20.9%	21.4%	NA
No SR score	41.8%	37.4%	34.3%	34.1%	NA
**Mother’s education**					
Less than primary	0.7%	0.7%	0.6%	0.6%	0.6%
Primary/Basic	26.3%	26.6%	24.8%	23.4%	24.7%
Secondary	50.7%	51%	51.5%	52%	55.6%
Tertiary or higher	22.3%	21.7%	23.1%	24%	19.1%
**Residence by region**					
Coast	56.3%	58.3%	57.6%	58.0%	56.4%
Highlands	36.4%	33.9%	34.3%	34.1%	35.7%
Amazon	7.4%	7.9%	8.1%	7.9%	7.9%
**Residence by area**					
Urban	49.9%	51.3%	52.1%	51.9%	63.2%
Rural	50.1%	48.7%	47.9%	48.1%	36.8%
**Maternal age**					
≤19 years	17.5%	16.6%	16%	15.1%	16.3%
20–29 years	50.6%	50.7%	50.5%	50.7%	53.1%
30–39 years	28.1%	28.9%	29.5%	30.1%	27.5%
≥40 years	3%	3.1%	3.3%	3.4%	3.1%
No data	0.8%	0.7%	0.7%	0.7%	NA

a ENDI, National Survey on Child Malnutrition; NA, not applicable.

b Nationally representative survey data on children under 2 years of age.

c SR, social registry poverty score based on a proxy means test using a statistical model based on living conditions to project consumption. Within the administrative system, individuals without an SR score are generally classified as not poor, although misclassification is possible. Comparable data were not available for the ENDI survey.

## How often have they been followed up?

SUUSEN integrates data on a daily basis: for the ENBIN cohort, data were extracted quarterly and harmonized into 6-month age intervals (0–5, 6–11, 12–17, and 18–23 months, inclusive). Within each interval, the most recent valid anthropometric measurement recorded during routine care was retained for analysis.

Follow-up is conditional on contact with public services. Anthropometric data are obtained primarily through scheduled ‘well-child’ visits at Ministry of Public Health primary care centres, supplemented by information from state-run home-visiting programmes, government childcare centres, and cash transfer monitoring systems. Children may contribute data intermittently across age intervals depending on service attendance: absence in one interval therefore does not necessarily indicate permanent loss to follow-up.

Coverage declines with age. For example, among children born between 2020 and 2023, 529 382 had at least one recorded check-up before 6 months of age, whereas 340 081 contributed data in the 18–23-month interval. Attrition may reflect migration, transition to private healthcare providers, irregular attendance at public facilities, or death; these mechanisms cannot be fully distinguished within the current administrative structure. Comparisons with ENDI indicate differential representation of some socioeconomic, geographic, and ethnic groups within ENBIN. In addition, follow-up itself may be socially patterned: children from higher-income urban households may increasingly receive care in the private sector, whereas migration, mobility and barriers to service access may reduce follow-up among some marginalized populations.

Because data were extracted in March 2024, follow-up to 24 months is complete only for children born in 2020 and 2021. For those born in 2022, follow-up at 18–23 months is available only for children born early in the year, and for those born in 2023, follow-up is limited to younger age intervals. Estimates at older ages therefore reflect children retained in the public health system and should not be interpreted as fully population-representative without triangulation with nationally representative survey data.

## What has been measured?

ENBIN provides linked population-based administrative data on birth conditions, anthropometry, socioeconomic status, and exposure to health and social protection services (Table 1).

**Table 1:** Data sources and information available in ENBIN cohort database.^a^

Data source	Variables collected
Birth registry	• 1 and 5-minute Apgar scores • weight and length at birth • gestation weeks at birth • type of delivery • parity • pregnancy outcomes • maternal education • ethnicity (self-identified) • municipality of residence • maternal age • maternal nationality • early or delayed clamping • breastfeeding within one hour of birth • early skin-to-skin contact
Ministry of health	• Attendance at prenatal and well-child check-ups • length and weight at each well-child check-up • *Z*-scores based on WHO standards • municipality of residence
Ministry of human development	• Access to home-visiting services • access to government-run childcare centres • access to government-run cash transfers
Social registry	• Municipality of residence • poverty status (based on the national proxy-means test)
Civil registry	• Municipality of residence • date of birth • sex • mother’s identity number • father’s identity number • date of birth registry • nationality
Constructed variables by SUUSEN	• Integrated data for the variables shown above • date of analysis • flags of unexpected anthropometric measurements

a SUUSEN—Sistema Unificado y Universal de Seguimiento Nominal (Unified and Universal Nominal Tracking System).

### Birth characteristics

Birth registration records provide basic demographic information, including sex, date of birth, nationality, ethnicity (self-identified at registration), municipality of residence, and anonymized parental identifiers, clinical information including birthweight, length at birth, gestational age, 1- and 5-minute Apgar scores, type of delivery, parity, maternal age and education, early breastfeeding, skin-to-skin contact, and umbilical cord clamping practices [15, 16].

Prematurity was defined as birth before 37 completed weeks of gestation [17]. Small-for-gestational-age (SGA) was defined using gestational-age-specific birthweight standards applied within the national registry system [17, 18]. Caesarean section was defined based on recorded mode of delivery.

### Anthropometry and growth indicators

Anthropometric measurements (weight and length/height) were obtained from routine well-child visits conducted in Ministry of Public Health facilities. Z-scores for length-for-age, weight-for-length, and BMI-for-age were calculated using WHO Child Growth Standards [5, 19]. Implausible anthropometric values were excluded using WHO-recommended biological plausibility criteria [19]. Where multiple measurements were recorded within a 6-month interval, the most recent valid measurement was retained.

For descriptive analyses, stunting was defined as length-for-age *z*-score < –2, wasting as weight-for-length *z*-score < –2, and overweight/obesity as BMI-for-age *z*-score > +2 [5, 19]. These indicators are widely used for international comparisons and monitoring of child nutritional status [4, 9].

### Socioeconomic status and services exposure

Socioeconomic status was derived from the SR, which assigns a poverty classification based on a proxy means test incorporating household living conditions and predicted consumption.

Data from the Ministry of Human Development capture exposure to government social protection programmes, including home-visiting services, attendance at government childcare centres, and receipt of cash transfers such as the Bono de Desarrollo Humano and Bono 1000 Días, the latter specifically designed to reduce childhood stunting. Health service utilization data include attendance at prenatal and well-child consultations, as well as indicators of timely birth registration.

Urban–rural classification and regional residence are based on official municipal designations recorded within the linked administrative registries. Because residence and ethnicity are derived from routine records, some degree of misclassification is possible, particularly in mobile or marginalized populations [10, 20, 21].

## What has it found?

Cohort composition remained broadly stable across birth years (Table 2). The proportion classified as extremely poor increased from 12.3% in 2020 to 21.4% in 2023, while the proportion without a Social Registry score declined. This likely reflects expansion and updating of Social Registry coverage rather than abrupt changes in underlying poverty.

Comparison with the nationally representative ENDI 2022–2023 survey (Table 2) [8] indicates broad consistency in regional distribution but notable differences in urban–rural classification and ethnicity. ENBIN classifies a higher proportion of children as living in rural areas and appears to under-represent Indigenous, Montubio, and Afro-Ecuadorian populations relative to ENDI. These patterns suggest that analyses using ENBIN may underestimate ethnic inequalities and over-represent rural populations. These discrepancies are consistent with previously documented ethnic inequalities in maternal and child health interventions in Ecuador and Latin America [10, 20, 21] and likely reflect structural features of administrative data systems, including differential service utilization, migration, and differences between administrative and household-based approaches to measuring residence and ethnicity. The impact of these differences on population-level estimates may be partly addressed through post-stratification weighting using external population benchmarks.

Birth characteristics (Supplementary Table 1) indicated high and slightly increasing caesarean section rates, reaching 49.6% among children born in 2022 and 2023. Early breastfeeding within the first hour of life was reported for approximately 90% of births. The proportion of infants with a 5-min Apgar score of 9–10 exceeded 91% across all birth years, suggesting generally favourable immediate neonatal conditions. The combined prevalence of prematurity or small-for-gestational age ranged from 14.2% to 15.4%, consistent with regional evidence on preterm birth and foetal growth restriction in low- and middle-income settings [17, 18].

Anthropometric indicators across the first 2 years of life are shown in Table 3. Denominators reflect children with at least one valid anthropometric measurement in each interval. Among children born in 2021, stunting prevalence increased from 12.9% at 0–5 months to 26.3% at 18–23 months. This pattern of increasing linear growth faltering during early childhood has been described in other longitudinal studies in low- and middle-income countries [5, 22, 23]. Wasting prevalence remained relatively low (∼2.5–3.5%) and varied little by age, consistent with other international data [19]. Overweight/obesity was greatest at 6–11 months and showed a modest decline across successive birth years. The coexistence of early overweight with substantial levels of stunting illustrates the double burden of malnutrition observed in Ecuador and other middle-income countries [11, 12].

**Table 3:** Prevalence (%) of malnutrition indicators during 24-month follow-up, stratified into 6-monthly intervals.^a^

Malnutrition indicator	Born in 2020 (*n* = 272 188)	Born in 2021 (*n* = 256 968)	Born in 2022 (*n* = 251 767)	Born in 2023 (*n* = 238 866)
**Children with follow-up at 0-5 months**	***n* = 104 354**	***n* = 117 321**	***n* = 148 365**	***n* = 159 342**
Stunting	12.9%	12.9%	14.1%	14.2%
Wasting	3.2%	3.2%	3.2%	3.4%
Overweight/obese	6.4%	6.5%	6.2%	6.1%
**Children with follow-up at 6-11 months**	***n* = 111 402**	***n* = 123 628**	***n* = 152 017**	***n* = 119 748**
Stunting	13.2%	14.0%	15.5%	13.9%
Wasting	2.5%	2.6%	2.8%	2.7%
Overweight/obese	9.6%	9.2%	8.5%	7.4%
**Children with follow-up at 12-17 months**	***n* = 105 240**	***n* = 129 435**	***n* = 146 930**	***n* = 32 349**
Stunting	17.9%	20.9%	22.3%	16.9%
Wasting	2.8%	3.0%	3.3%	2.7%
Overweight/obese	8.7%	7.9%	7.0%	5.2%
**Children with follow-up at 18-23 months**	***n* = 112 805**	***n* = 131 123**	***n* = 96 153**	**NA**
Stunting	21.6%	26.3%	25.4%	NA
Wasting	2.5%	2.7%	2.6%	NA
Overweight/obese	8.1%	7.3%	6.3%	NA

a Due to the March 2024 data cut-off, follow-up data at older ages for children born more recently is missing—for children born in 2022, follow-up is available only for those born in the first months of the year at 18–23-months, while for children born in 2023, only those born in the first 3 months have follow-up data for the 12–17-month period.

**Table 4:** Potential future data sources for linkage with the ENBIN cohort.

Data linkage at the nominal (individual) level	Data linkage at the aggregated (geographic) level
Civil Registry digital death certificates	2022 Population and dwelling census (municipal/provincial data on housing, access to services)
Ministry of Public Health and social insurance records (e.g. hospitalizations, outpatient visits, disease registries)	Annual economic and environmental statistics of municipal governments (local public service provision)
Ministry of Education data (e.g. school enrolment, standardized test scores)	Climate and environmental data (e.g. from National Institute of Meteorology, National Risk Management Secretary)
Social security system data (parental formal employment history)	

Coverage of health and social protection services improved across successive cohorts (Supplementary Table 2). For children born in 2023, compliance with recommended prenatal and early well-child visits was higher than in earlier cohorts. Coverage of the conditional Bono 1000 Días cash transfer and home-visiting programmes also increased over time. Conditional and unconditional cash transfer programmes have been associated with improvements in child nutrition and health outcomes in other settings [24–26]. These temporal patterns are consistent with progressive implementation of ENECSDI. Exploratory multivariable analyses (not shown) are consistent with established evidence linking social disadvantage and adverse birth conditions with poorer early-life growth outcomes [18, 27–29], illustrating the potential of ENBIN for joint analyses of biological and social determinants.

Overall, ENBIN demonstrates the utility of linked administrative data for characterizing early-life health, growth trajectories and social inequalities at national scale. The findings highlight patterns in early-life nutrition and changes in service coverage over time, consistent with national and regional evidence [1, 2, 10, 20] on social inequalities in child health. Taken together, these features underscore the value of ENBIN as a platform for analyzing the joint effects of biological and social determinants in early life and for informing policies aimed at reducing inequalities in child health.

## What are the main strengths and weaknesses?

The ENBIN cohort is a large, national administrative birth cohort including over one million children born in Ecuador between 2020 and 2023. ENBIN represents the largest population-based birth cohort assembled in Ecuador and provides national-scale longitudinal data focused on early childhood. Its principal strength is the individual-level integration of civil registration, health service, social protection, and poverty registry data, enabling longitudinal follow-up from birth to 2 years and joint analysis of biological and social determinants of nutrition. The scale of the cohort provides substantial statistical power for subgroup analyses and offers strong potential for future life-course linkage to mortality, education, and hospitalization records (examples provided in Table 4), as well as environmental and climate data [30, 31].

However, ENBIN is not fully population-representative. Although birth registration coverage is high, follow-up anthropometric data depend on contact with public health services and decline with age. Differential service use, migration, private-sector care, and incomplete recording of residence and ethnicity may introduce selection and misclassification bias, particularly for urban coastal, rural Amazon, and ethnic minority populations.

Anthropometric measurements are collected in routine clinical settings and may vary in precision, and socioeconomic status is derived from administrative poverty scores that are missing for a proportion of children.

Several processes may affect representativeness, including differential initial capture, socially patterned service use, migration, intermittent follow-up, item-level missingness and administrative gaps. Their importance will depend on the specific research question. Future analyses should therefore characterize predictors of observation and attrition and assess whether these vary by ethnicity, region, socioeconomic status, and service use. Where appropriate, post-stratification or inverse probability weighting, multiple imputation, and sensitivity analyses can be used to assess and partly address resulting selection and missing-data biases [14].

Overall, ENBIN should therefore be interpreted as a large and highly informative longitudinal administrative resource that complements nationally representative survey data. Its scale, linkage structure, and temporal depth provide a strong platform for analyzing early-life health and inequalities, while its limitations are transparent and amenable to appropriate methodological handling.

## Can I get hold of the data? Where can I find out more?

ENBIN data were obtained through a formal data-sharing agreement with the Technical Secretariat ‘Ecuador Crece sin Desnutrición Infantil’ (STECSDI), formerly responsible for ENECSDI and now subsumed within the Ministry of Human Development. The agreement permits academic and non-commercial use. Under the terms of this agreement, ENBIN cohort data are held by the Social and Environmental Determinants of Health Inequalities (SEDHI) research unit housed at Universidad Internacional del Ecuador (UIDE). The unit welcomes collaboration with researchers from other national and international institutions. Interested parties are encouraged to contact the SEDHI unit for further information on how to access ENBIN data for research purposes. Alternatively, researchers interested in accessing the SUUSEN dataset could contact the Ministry of Human Development of Ecuador directly, with access provided subject to formal request procedures.

Profile in a nutshell

- **Why was the cohort set up/unique features:** the ENBIN cohort was established to address the lack of longitudinal, population-scale data on early-life determinants of child growth in Ecuador by leveraging a national administrative data linkage platform developed under the ENECSDI strategy.
- **Location, baseline years, and participants:** ENBIN includes 1 019 789 children born between 1 January 2020 and 31 December 2023, representing over 95% of births nationally in Ecuador, and is structured as four annual birth cohorts with follow-up from birth to 24 months.
- **Follow-up and attrition:** follow-up is based on routinely collected administrative data and is conditional on engagement with public health and social services; coverage declines with age, with 529 382 children contributing data before 6 months and 340 081 at 18–23 months.
- **Main data collected:** the cohort integrates individual-level data on birth characteristics (e.g. gestational age, birthweight, Apgar scores), anthropometry (weight and length/height), socioeconomic indicators (poverty status, maternal characteristics), health service utilization, and exposure to social protection programmes.
- **Collaboration and data access:** ENBIN provides a large-scale longitudinal platform for analyzing growth trajectories, health inequalities, and early-life exposures. Data are available for collaborative research through the SEDHI Unit at UIDE or via formal request to the Ministry of Human Development in Ecuador.

## Ethics approval

These data were collected for administrative and surveillance purposes and were provided by the STECSDI in a fully anonymized format, with no personally identifiable information, direct identifiers (such as names or ID numbers), or indirect identifiers (such as detailed geographic coordinates) included. This profile does not involve the collection of new data, contact with human participants, or the use of identifiable information. In accordance with national regulations and institutional policies governing the use of secondary, de-identified data, this analysis of ENBIN did not require formal ethical approval. Data provision and handling followed STECSDI’s internal protocols and Ecuadorian regulations governing public-sector data sharing and research use. The data were accessed in compliance with Ecuadorian regulations governing public sector data sharing and research use.

## Supplementary Material

dyag221_Supplementary_Data

## Data Availability

See ‘Can I get hold of the data?’ above.
