# Supplementary material for: Cohort Profile: The Ecuadorian National Birth and Infant Nutrition cohort
Source: Int J Epidemiol. 2026 Oct 4;55(5):dyag221. doi: 10.1093/ije/dyag221 (PMC13633846; doi:10.1093/ije/dyag221)
Supplement: Supplementary file 1 [file dyag221_supplementary_data.docx]

**Supplementary Table 1.** **Percentages (%) of birth conditions in the ENBIN cohort**

| Characteristic | Born in 2020 | Born in 2021 | Born in 2022 | Born in 2023 |
| --- | --- | --- | --- | --- |
| Caesarean section | 47.6% | 48.8% | 49.6% | 49.6% |
| Breastfed within first hour | 89.1% | 89.7% | 90.0% | 90.6% |
| APGAR Score 9-10 (at 5 min) | 92.4% | 92.0% | 92.0% | 91.8% |
| Adverse birth outcomes | | | | |
| Prematurity | 6.7% | 6.9% | 7.4% | 7.6% |
| Small for Gestational Age (SGA) | 8.4% | 8.0% | 8.3% | 8.6% |
| Premature or SGA | 14.3% | 14.2% | 15.0% | 15.4% |
| ***Participants*** | ***238 707*** | ***229 974*** | ***228 053*** | ***203 247*** |

**Supplementary Table 2.** **Percentages (%) of children with access to services during 24 months follow-up, stratified into 6-monthly intervals.**

| Service | Born in 2020 | Born in 2021 | Born in 2022 | Born in 2023 |
| --- | --- | --- | --- | --- |
| ***Children followed up at 0-5 months*** | ***n=272 188*** | ***n=256 968*** | ***n=251 767*** | ***n=238 866*** |
| Prenatal check-up compliance | 34.9% | 41.6% | 46.4% | 51.5% |
| Well-Child check-up compliance | 37.2% | 41.4% | 48.9% | 54.3% |
| Bono 1000 Días | 0.0% | 0.0% | 9.7% | 24.4% |
| Other cash transfers | 13.3% | 15.6% | 19.3% | 17.2% |
| Childcare centres (CDI) | 0.4% | 0.8% | 0.2% | 0.0% |
| Home-Visiting (CNH) | 5.0% | 7.5% | 12.1% | 16.3% |
| Timely birth registration  (<45 days) | 45.9% | 59.4% | 64.0% | 71.6% |
| ***Children followed up at 6-11 months*** | ***n=271 542*** | ***n=256 349*** | ***n=251 024*** | ***n=191 646*** |
| Prenatal check-up compliance | 34.9% | 41.6% | 46.4% | 50.9% |
| Well-Child check-up compliance | 30.8% | 35.8% | 45.0% | 52.1% |
| Bono 1000 Días | 0.0% | 0.0% | 9.7% | 24.4% |
| Other cash transfers | 21.2% | 17.4% | 19.4% | 16.9% |
| Childcare centres (CDI) | 1.7% | 1.8% | 0.1% | 0.0% |
| Home-Visiting (CNH) | 15.6% | 18.3% | 24.3% | 25.7% |
| Timely birth registration  (<45 days) | 45.8% | 59.3% | 64.0% | 70.7% |
| ***Children followed up at 12-17 months*** | ***n=271 363*** | ***n=256 135*** | ***n=250 793*** | ***n=70 204*** |
| Prenatal check-up compliance | 34.9% | 41.6% | 46.4% | 50.1% |
| Well-Child check-up compliance | 28.9% | 35.1% | 44.3% | 49.3% |
| Bono 1000 Días | 0.0% | 0.0% | 10.0% | 25.4% |
| Other cash transfers | 21.9% | 20.3% | 18.8% | 17.1% |
| Childcare centres (CDI) | 6.9% | 7.5% | 7.0% | 2.0% |
| Home-Visiting (CNH) | 20.6% | 21.4% | 25.3% | 27.4% |
| Timely birth registration  (<45 days) | 45.8% | 59.3% | 64.0% | 69.0% |
| ***Children followed up at 18-23 months*** | ***n=271 235*** | ***n=255 971*** | ***n=198 394*** | ***NA*** |
| Prenatal check-up compliance | 34.9% | 41.6% | 45.5% | NA |
| Well-Child check-up compliance | 28.7% | 35.5% | 43.2% | NA |
| Bono 1000 Días | 0.0% | 0.0% | 7.8% | NA |
| Other cash transfers | 23.4% | 20.3% | 18.3% | NA |
| Childcare centres (CDI) | 12.0% | 12.4% | 10.5% | NA |
| Home-Visiting (CNH) | 22.0% | 22.4% | 24.2% | NA |
| Timely birth registration  (<45 days) | 45.8% | 59.3% | 63.4% | NA |

Due to the March 2024 data cut-off, follow-up data at older ages for children born more recently is missing - for children born in 2022, follow-up is available only for those born in the first months of the year at 18–23-months, while for children born in 2023, only those born in the first 3 months have follow-up data for the 12–17-month period.
